# A microfluidic system that replicates pharmacokinetic (PK) profiles in vitro improves prediction of in vivo efficacy in preclinical models

**DOI:** 10.1371/journal.pbio.3001624

**Published:** 2022-05-26

**Authors:** Dharaminder Singh, Sudhir P. Deosarkar, Elaine Cadogan, Vikki Flemington, Alysha Bray, Jingwen Zhang, Ronald S. Reiserer, David K. Schaffer, Gregory B. Gerken, Clayton M. Britt, Erik M. Werner, Francis D. Gibbons, Tomasz Kostrzewski, Christopher E. Chambers, Emma J. Davies, Antonio Ramos Montoya, Jacqueline H. L. Fok, David Hughes, Kristin Fabre, Matthew P. Wagoner, John P. Wikswo, Clay W. Scott

**Affiliations:** 1 CN Bio Innovations Limited, Cambridge, United Kingdom; 2 Oncology Safety, Clinical Pharmacology & Safety Sciences, BioPharmaceuticals R&D, AstraZeneca, Boston, Massachusetts, United States of America; 3 Bioscience, Oncology R&D, AstraZeneca, Cambridge, United Kingdom; 4 Bioscience, Oncology R&D, AstraZeneca, Boston, Massachusetts, United States of America; 5 Department of Physics and Astronomy and the Vanderbilt Institute for Integrative Biosystems Research and Education, Nashville, Tennessee, United States of America; 6 DMPK, Oncology R&D, AstraZeneca, Boston, Massachusetts, United States of America; 7 MPS Center of Excellence, Clinical Pharmacology & Safety Sciences, BioPharmaceuticals R&D, AstraZeneca, Boston, Massachusetts, United States of America; 8 Departments of Biomedical Engineering and Molecular Physiology and Biophysics, Vanderbilt University, Nashville, Tennessee, United States of America; Stanford University, UNITED STATES

## Abstract

Test compounds used on in vitro model systems are conventionally delivered to cell culture wells as fixed concentration bolus doses; however, this poorly replicates the pharmacokinetic (PK) concentration changes seen in vivo and reduces the predictive value of the data. Herein, proof-of-concept experiments were performed using a novel microfluidic device, the Microformulator, which allows in vivo like PK profiles to be applied to cells cultured in microtiter plates and facilitates the investigation of the impact of PK on biological responses. We demonstrate the utility of the device in its ability to reproduce in vivo PK profiles of different oncology compounds over multiweek experiments, both as monotherapy and drug combinations, comparing the effects on tumour cell efficacy in vitro with efficacy seen in in vivo xenograft models. In the first example, an ERK1/2 inhibitor was tested using fixed bolus dosing and Microformulator-replicated PK profiles, in 2 cell lines with different in vivo sensitivities. The Microformulator-replicated PK profiles were able to discriminate between cell line sensitivities, unlike the conventional fixed bolus dosing. In a second study, murine in vivo PK profiles of multiple Poly(ADP-Ribose) Polymerase 1/2 (PARP) and DNA-dependent protein kinase (DNA-PK) inhibitor combinations were replicated in a FaDu cell line resulting in a reduction in cell growth in vitro with similar rank ordering to the in vivo xenograft model. Additional PK/efficacy insight into theoretical changes to drug exposure profiles was gained by using the Microformulator to expose FaDu cells to the DNA-PK inhibitor for different target coverage levels and periods of time. We demonstrate that the Microformulator enables incorporating PK exposures into cellular assays to improve in vitro–in vivo translation understanding for early therapeutic insight.

## Abstract 2

Conventionally, in vitro model systems poorly replicate the pharmacokinetic concentration changes seen in vivo. This study demonstrates the capability of a microfluidic device, the Microformulator, to replicate pharmacokinetic profiles faithfully in vitro for better predictability of preclinical models.

## Introduction

The challenges faced by the pharmaceutical industry in developing novel medicines, including attrition rates from early discovery to clinical validation, have been well described [1]. Kola and Landis [2] highlighted the most common causes of R&D attrition as being a lack of efficacy and/or safety and suggested that more attention be paid to reducing toxicity risks, improving preclinical models and demonstrating adequate proof of mechanism and proof of concept in the clinic. It can be hypothesised that the introduction of novel predictive preclinical testing techniques could improve the speed and reduce attrition in developing therapeutic compounds.

**Fig 1:**
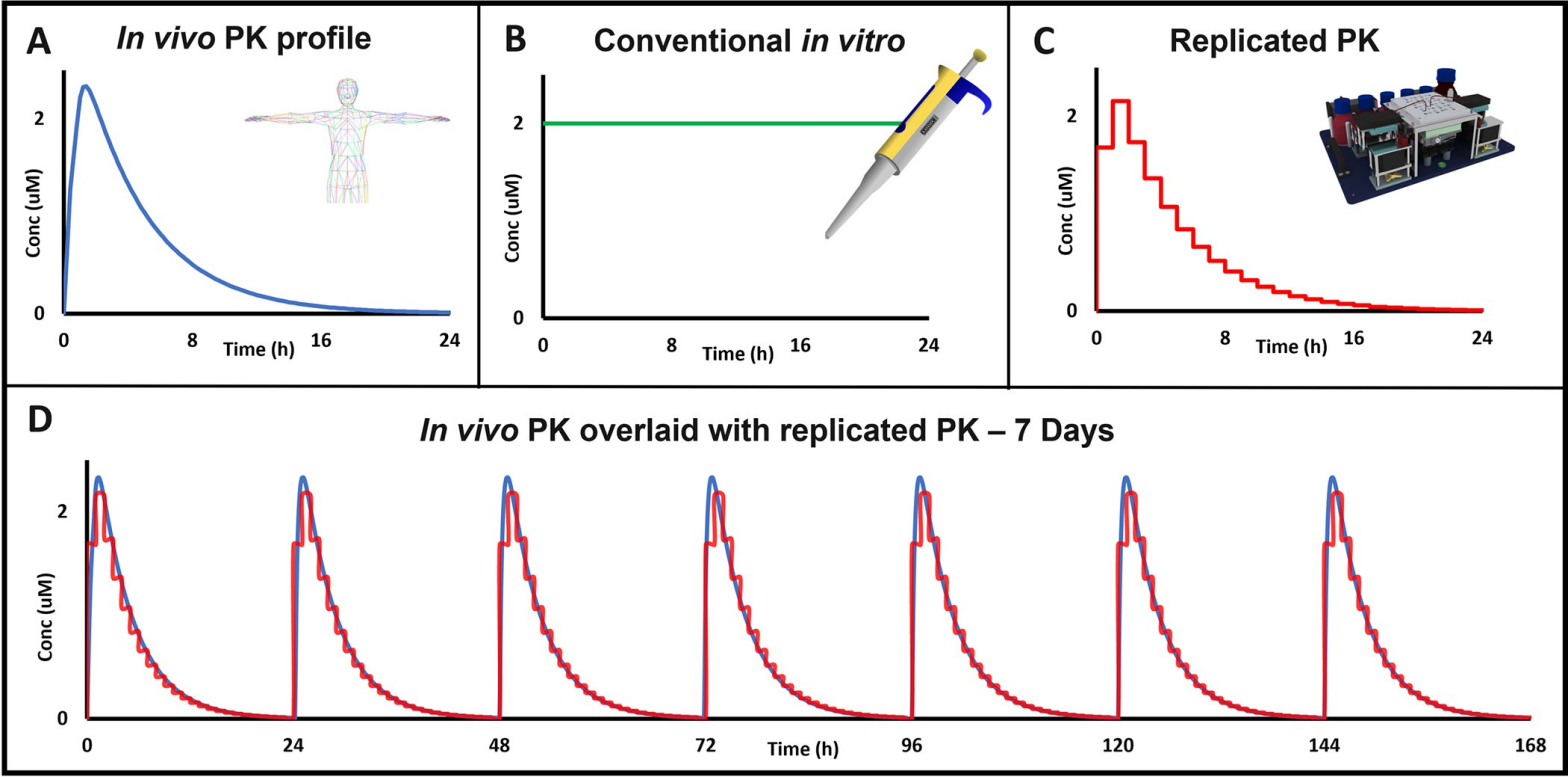
Improving translation of in vitro assays requires incorporating in vivo drug exposure profiles. (A) In contrast to the dynamic peak and trough exposure profile that occurs in vivo reflecting drug absorption, distribution, and elimination, most in vitro assays (B) use a static, fixed bolus concentration of drug for the duration of the assay. (C) The Microformulator can replicate a PK profile and can (D) repeat the profile for multiple days/weeks to enable PK-PD-efficacy testing in vitro to determine the degree of target inhibition and the duration of inhibition required for a biological response. PD, pharmacodynamics; PK, pharmacokinetic.

A wide variety of in vitro preclinical models are available, from genetically engineered cell lines to patient-derived organoids [3–5]. These models are utilised for purposes including target validation studies, testing the efficacy of novel therapeutic compounds and gaining insight into potential clinical indications for particular targets [6]. These in vitro efficacy studies are routinely performed with fixed concentrations of compound, dosed as a bolus at the start of the incubation and if required by the protocol refreshed every few days afterwards. Within the human body, however, the concentration of a compound changes continuously over time as the drug is absorbed, distributed, metabolised, and excreted. This concentration change, known as the pharmacokinetic (PK) profile, is important in the drug development process as the efficacy of a compound is determined by the free concentration available at the site of action and hence is a function of the PK profile [7]. Being able to incorporate PK profiles into in vitro studies will enable exploration of the relationship between PK and efficacy, potentially revealing therapeutic insights earlier in the drug discovery process than is currently possible (Fig 1).

A number of methodologies and devices have been described, which aim to replicate PK profiles in vitro. Both dilution and macroscale hollow fibre diffusion techniques have been used to dynamically modulate antibiotic concentrations within bacterial cultures [8–12]. A small number of simple microfluidic systems have explored passive diffusion [13], gravity-driven flow [14], and chip-based microfluidics to achieve time-dependent changes in drug concentration [15–18]. All of these systems, however, are limited in their use as they often have low throughput (limited number of wells/test conditions), provide little control over compound concentration changes (limited number of concentration changes/limited concentration range), lack integration with standard in vitro cell culture systems/models, or are restricted to short-term experiments.

Herein, we describe a microfluidic device designed to change the concentration of a test compound within individual wells of a standard microtiter plate over time, to create a predetermined and distinct replicated PK profile in each well. This is achieved through the addition and removal of cell culture medium or test compound at discrete intervals, creating stepwise changes in the concentration of the test compound over a wide concentration range and capable of supporting multiweek experiments. One or 2 different compounds can be administered simultaneously with independent PK profiles, thereby enabling the evaluation of drug combinations. Experiments are described using this instrument to explore the relationship between PK, pharmacodynamics (PD), and efficacy of oncology compounds in vitro and to compare the results to those obtained in vivo.

## Results

### Replicating PK profiles—The Microformulator

**Fig 2:**
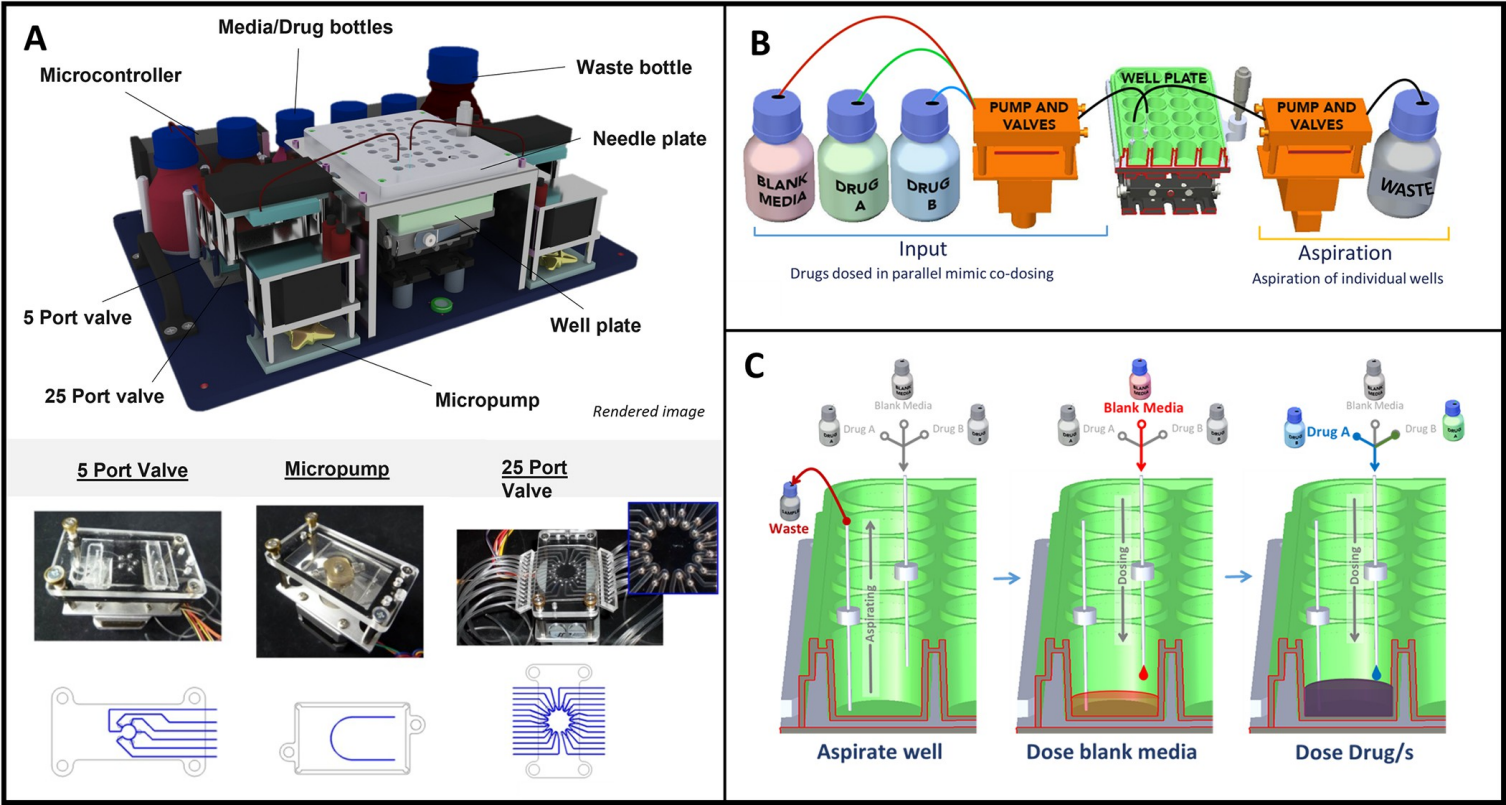
The Microformulator, a microfluidic device used to accurately dose and aspirate media from individual wells of a standard microtiter plate. (A) 3D render of the Microformulator device showing various component parts. Photographs of a micropump and valves and the fluidic paths within those devices are shown below. (B) Schematic representation of the Microformulator setup, split into an input side and an aspiration side. During each sweep/cycle media is pumped from input bottles through a series of valves into the well plate. On the aspiration side, media is drawn from the wells through a 25-port valve and pumped into waste. (C) Schematic representation of media addition and removal from each well on a microtiter plate using the Microformulator. Blank media and media containing drug/s are dosed into the well from different bottles to regularly change the concentration within the well.

The Microformulator is a microfluidic device comprised of valves, pumps, and fluid reservoirs under computer control that enable mixing microliter volumes of cell culture media and drug solutions and delivering these mixtures to individual wells within a microtiter plate (Fig 2). A replicate set of valves and pumps aspirates fluid out of the wells; coordinating the aspiration and dispensing enables changing the drug concentration in each well. A controller is programmed to generate a defined drug exposure profile over a set time interval (24-hour) cycle using predefined intervals of drug/media refresh to achieve the desired time-dependent change in drug concentrations [19]. Each well is individually addressable, and 1 or 2 compounds can be administered simultaneously, each with its own PK profile. The unit operates within a standard cell culture incubator (S3 Fig) and can continue delivering compounds for experiments lasting multiple weeks if desired. The ability of the fluidics system to change drug concentrations was verified using fluorescein as the test compound and performing 3X steps of increasing and decreasing concentrations over a 3,000-fold concentration range (S1 Fig), which is sufficient to characterise pharmacological exposure response profiles. Experiments also were performed with 2 of the pharmacological agents used in this study to confirm the delivery of the targeted concentrations by the Microformulator (S1 Fig).

It is widely reported that preclinical strategies for the evaluation of cancer therapeutics are suboptimal; therefore, several experiments using oncology compounds targeting different biological pathways were performed to evaluate the utility of this device [20–22]. These experiments determine whether replicating PK profiles with the Microformulator in cell-based assays can achieve efficacious responses similar to that seen in vivo. The first study compared the efficacious response profile using Microformulator-generated PK exposure to that obtained with traditional fixed concentration bolus dosing. For this study, the ERK1/2 inhibitor AZD0364 was tested in both a sensitive and nonsensitive cell line pair, grown in 24-well microtiter plates. A second study was performed to determine whether replicating PK profiles with the Microformulator in cell-based assays can achieve efficacious responses similar to that seen in vivo. The Microformulator was used in 96-well format to replicate murine PK profiles of Poly(ADP-Ribose) Polymerase (PARP) inhibitor olaparib and a DNA-dependent protein kinase (DNA-PK) inhibitor AZD7648 as single agents and in combination.

### Comparing the biological response to fixed concentration versus Microformulator-generated PK dosing

The RAS-RAF-MEK1/2-ERK1/2 (RAS/MAPK) signalling pathway is frequently dysregulated in human cancers, often by mutations in *BRAF* or *RAS* genes and therefore is an important node for development of novel oncology compounds. In a recent publication, the ERK 1/2 inhibitor AZD0364 was profiled across different in vivo xenograft models [23–25]. Treatment of the non-small cell lung cancer (NSCLC) Calu-6 *KRAS* mutant xenograft model with AZD0364 at 50 mg/kg once daily (QD) for 21 days resulted in tumour regressions. In comparison, the A549 NSCLC *KRAS* mutant xenograft model was less sensitive to the same dose of AZD0364 reaching only partial tumour growth inhibition (TGI) [23]. The purpose of this experiment was to use the Microformulator to determine if this difference in response of the 2 *KRAS* mutant xenografts to AZD0364 could be replicated in an in vitro system and, if so, to compare results to traditional fixed concentration bolus dosing.

**Fig 3:**
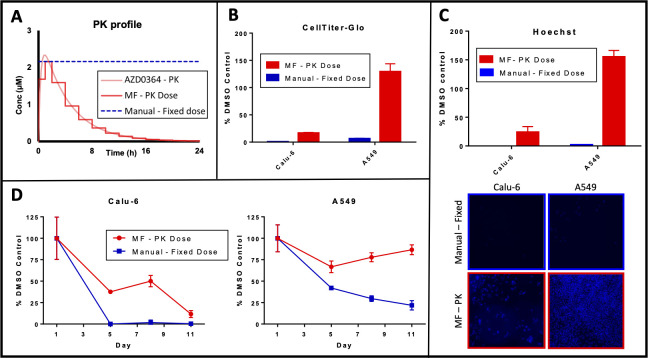
ERK1/2 inhibitor AZD0364 in vivo PK profile replicated on the Microformulator in vitro demonstrates differential activity in *KRAS* mutant cell lines. (A) In vivo PK profile of AZD0364 (50 mg/kg QD) in murine models (light red) was replicated in vitro using the Microformulator (deep red), where each dosing step represents a 1- or 2-hour interval. A manual fixed dose (equivalent to maximum dose delivered by Microformulator) was performed alongside the Microformulator in typical in vitro conditions with media and drug replenishment every 72 hours. (B, C, D) Growth inhibitory response (B), determined through CellTiter-Glo endpoint ATP quantification normalised to DMSO control on D6. Data represent mean ± SEM of 3 technical replicates. Hoechst DNA analysis (C) performed on images normalised to DMSO control on D11. Data represent mean ± SEM of 3 images. Growth inhibitory response further determined (D) from confluency analysis of brightfield images normalised to DMSO controls, of *KRAS* mutant cells Calu-6 and A549 to AZD0364 delivered by the Microformulator each day over 11 days of treatment (red) or as a fixed dose (2.16 μM) (blue). Data represent mean ± SD of 3 technical replicates. Underlying data can be found in S1 Data. PK, pharmacokinetic.

Calu-6 and A549 cell lines were cultured in the Microformulator for 11 days. The Microformulator was programmed to replicate the in vivo PK profile of AZD0364 with a 50-mg/kg QD schedule. The replicated Microformulator PK profile operated in 1- and 2-hour steps, resulting in 13 steps over 24 hours (Fig 3A). For both cell lines the Microformulator PK profile of AZD0364 was compared to a fixed bolus dose of 2.16 μM (matched to the highest concentration delivered by the Microformulator) and the equivalent, manually dosed, DMSO control. Brightfield microscopy images of both cell lines were captured over time up to 11 days post the initiation of treatment and used to determine cell confluency (Fig 3D). Cell confluency results were confirmed separately through ATP endpoint quantification and DNA analysis (Fig 3B and 3C).

The growth of more sensitive Calu-6 cell line was inhibited by both the Microformulator PK dose and the manual dose of AZD0364, with the manual dose showing greater response at the earlier time points. (Fig 3D). In contrast, the growth of less sensitive A549 cell line was significantly inhibited by the fixed bolus dose of AZD0364, whereas the Microformulator PK dose only weakly inhibited the growth of A549 cells over the duration of the experiment (Fig 3B–3D). These data indicate that the differences in cell line sensitivity is more accurately reflected in Microformulator PK doses of AZD0364 than the manual fixed concentration of AZD0364, when compared to the Calu-6 and A549 xenografts reported in vivo [23].

### Replicating in vivo PK for in vitro efficacy testing

**Fig 4:**
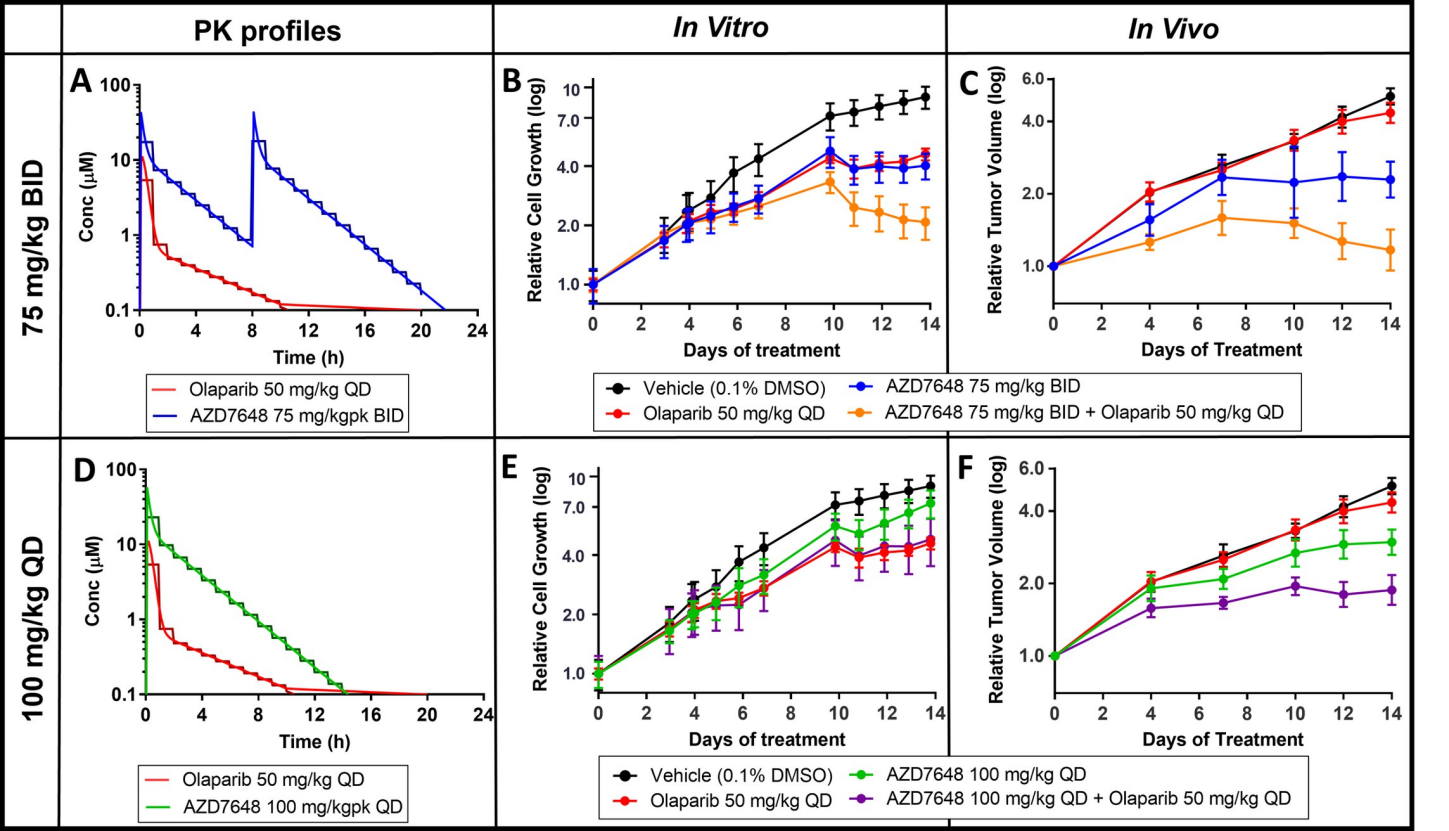
Olaparib and DNA-PK inhibitor AZD7648 in vivo PK profiles replicated using the Microformulator: comparing antiproliferative effect in vitro with in vivo results. **(A)** In vivo PK profiles of olaparib (50 mg/kg QD) and AZD7648 (75 mg/kg BID) (continuous line, red and blue) matched with Microformulator-generated PK profiles (stepped line, red and blue). (B) IncuCyte analyisis of FADU ATM KO cell growth inhibition effects of olaparib and AZD7648 (75 mg/kg BID) monotherapy or combination delivered by the Microformulator each day for 14 days. (C) TGI of FaDu ATM KO xenografts treated with olaparib and AZD7648 (75 mg/kg BID) as monotherapy and in combination (SCID mice, *n =* 4–9 day 14). Data represent geomean ± SEM (tumour start volume 0.26 cm^3^). (D) In vivo PK profiles of olaparib (50 mg/kg QD) and AZD7648 (100 mg/kg QD) (continuous line, red and green) matched with Microformulator-generated PK profiles (stepped line, red and green). (E) IncuCyte analyisis of FADU ATM KO cell growth inhibition effects of olaparib and AZD7648 (100 mg/kg QD) monotherapy or combination delivered by the Microformulator each day for 14 days. (F) TGI of FaDu ATM KO xenografts treated with olaparib and AZD7648 (100 mg/kg QD) as monotherapy and in combination (SCID mice, *n* = 8–9 day 14). Data represent geomean ± SEM (tumour start volume 0.26 cm^3^). Underlying data can be found in S2 Data. DNA-PK, DNA-dependent protein kinase; KO, knockout; PK, pharmacokinetic; TGI, tumour growth inhibition.

Mouse xenograft studies have shown that the DNA-PK inhibitor AZD7648 can enhance the efficacy of olaparib, a PARP1/2 inhibitor, in FaDu ATM knockout (KO) tumour models, since the loss of ATM has been shown to sensitise cells to DNA-PK inhibitor treatment [26,27]. An in vitro study was designed to determine whether this enhanced efficacy could be recapitulated using the Microformulator recreating PK exposure profiles from the mouse xenograft efficacy study (Fig 4). FaDu ATM KO cells were plated in a 96-well microtiter plate and then treated daily for 14 days with either olaparib mimicking a 50-mg/kg once daily (QD) profile (red), AZD7648 at 75 mg/kg twice daily (BID) (green) or 100 mg/kg QD (blue), or combinations of the 2 compounds (orange and purple). In this study, the Microformulator delivered compounds using 1-hour dosing intervals. The effect of drug treatments on cell growth was quantified using an Incucyte imaging system at various days throughout the study.

The Microformulator-delivered treatments of olaparib (red) or AZD7648 at 75 mg/kg BID (blue) gave partial suppression of tumour cell growth, whereas the combination of both agents gave significantly greater inhibition (orange) (Fig 4B). In a 2-week mouse xenograft study (Fig 4C), olaparib (red) had little effect whereas AZD7648 75 mg/kg BID (blue) gave partial suppression, and the combination of both agents gave superior inhibition of tumour growth (orange). The relative efficacy of AZD7648 75 mg/kg BID alone (blue) and in combination (orange and purple) delivered by the Microformulator to FaDu ATM KO cells was similar to that seen in the in vivo FaDu ATM KO tumour xenograft model. It is clear that the Microformulator-generated 100 mg/kg QD dose of AZD7648 (green) was less efficacious than the 75-mg/kg BID schedule (blue), results that again mirrored the in vivo findings. In addition, the-100 mg/kg QD combination with olaparib (purple) gave less tumour cell growth suppression than did the 75-mg/kg BID combination (orange). This was true for both the Microformulator study and mouse xenograft study. Thus, except for olaparib monotherapy being more efficacious in vitro than in vivo, the AZD7648 single agent results and combinations showed similar rank order effects in vivo and in vitro when using the Microformulator.

### Expanding PK/efficacy understanding

**Fig 5:**
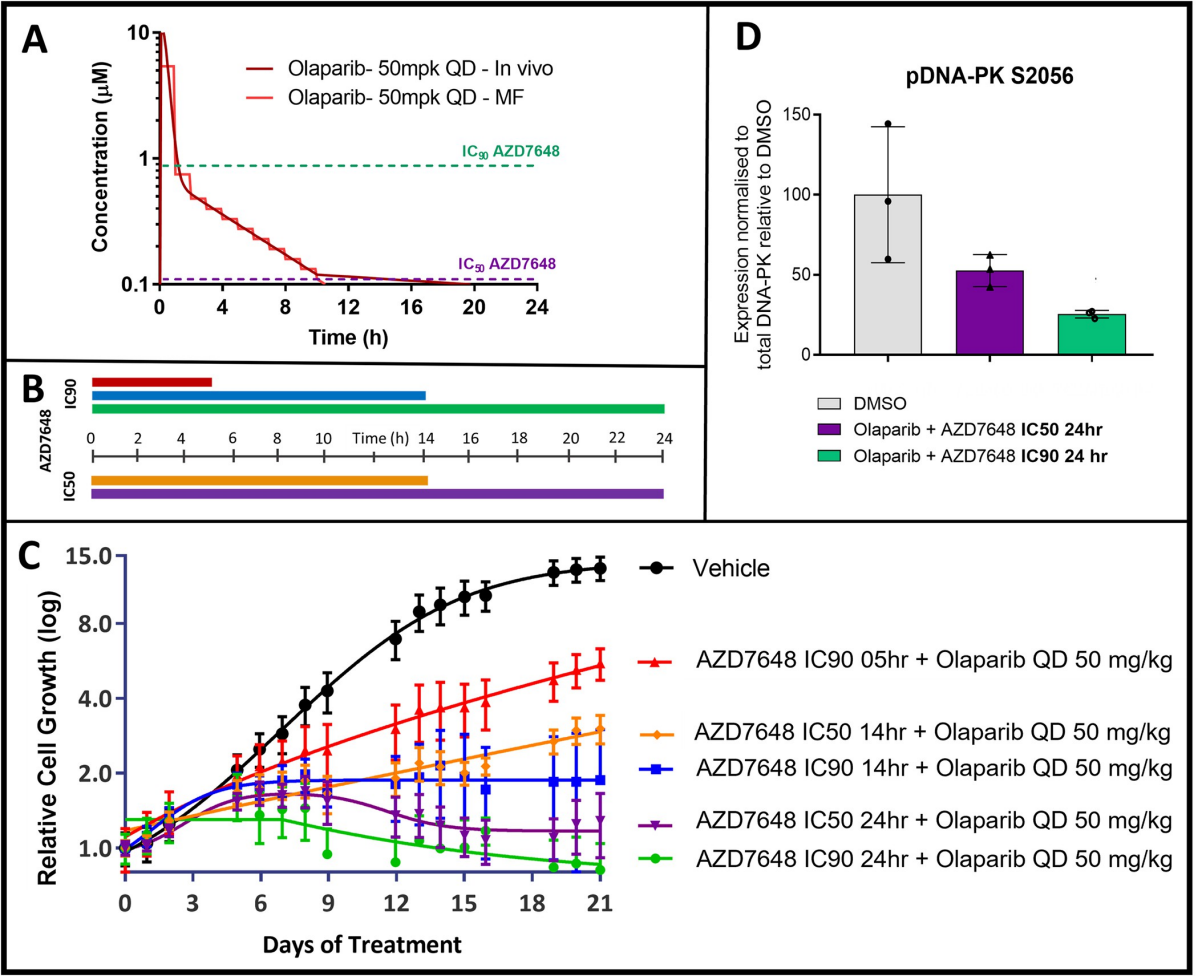
Investigating AZD7648 exposure parameters to maximise olaparib combination efficacy. **(A)** In vivo and Microformulator-replicated PK profile of olaparib (50 mg/kg QD) and fixed IC_90_ and IC_50_ concentrations for DNA-PK AZD7648. **(B)** Durations of daily exposure to AZD7648 at either IC_90_ or IC_50_, (colours matched to C). **(C)** Growth inhibitory response, determined from Incucyte analysis, of FaDu ATM KO cells to olaparib and AZD7648 treatment delivered by the Microformulator at fixed IC_90_ and IC_50_ concentrations for indicated durations throughout each 24-hour cycle over 3 weeks. Data represent mean ± SEM of 3 technical replicates. **(D)** FaDu ATM KO cells were treated for 24 hours with olaparib plus AZD7648 at either IC_50_ or IC_90_ and whole cell lysates were collected. Proteins were separated by SDS-PAGE and detected by western blotting. The band intensity of pDNA-PK S2056 was quantified and expression was normalised to the quantity of the respective total protein. Graphs represent mean expression ± SD for pDNA-PK relative to its expression in the DMSO control. Individual data points represent technical replicates. Underlying data can be found in S3 Data. DNA-PK, DNA-dependent protein kinase; KO, knockout; PK, pharmacokinetic.

Since the lower dose of AZD7648 given twice daily had greater combination efficacy than the higher dose given once per day, an additional study was designed to gain insight on how aspects of the AZD7648 PK profile influence combination efficacy. In this study, the Microformulator was used to deliver fixed concentrations of AZD7648 for different lengths of time to investigate the relative importance of the extent of target inhibition versus the duration of target inhibition. FaDu ATM KO cells were treated with modest and high concentrations (representing the IC_50_ and IC_90_ concentration) of AZD7648 at either 0.1 μM or 0.9 μM, to inhibit DNA-PK in cells in vitro (26) (Fig 5A and 5B). The cells were exposed to these concentrations for 5 hours, 14 hours, or 24 hours per day for 21 days while also receiving olaparib 50 mg/kg PK each day. The results from this study show that a short exposure with high target coverage of DNA-PK is insufficient for robust tumour cell growth inhibition: Longer exposure gives greater combination efficacy (Fig 5C). Likewise, at a similar exposure period, the higher target coverage gives greater efficacy. This is consistent with down-regulation of phosphorylated DNA-PK, a PD biomarker of DNA-PK inhibition, where the higher test concentration achieved greater suppression of DNA-PK activity (Fig 5D). Thus, maximal combination efficacy for FaDu ATM KO cells requires sustained, high inhibition of DNA-PK. Results also show that modest target coverage (IC_50_) of DNA-PK with a 14-hour exposure had greater efficacy than higher target coverage (IC_90_) at the shorter exposure.

## Discussion

Multiwell microtiter plates are the backbone of in vitro life sciences and drug discovery research. Enormous infrastructure exists to facilitate their use and includes liquid handling and signal detection instruments compatible with a wide range of cell types (primary cells, cell lines, and organoids), formats (monolayer, sandwich culture, spheroids, and 3D scaffold), and readouts (fluorescence, high content imaging, soluble and cell extracted biomarkers, and omics) [28]. However, these in vitro systems have limitations when replicating human biology, a fundamental one being that they allow only fixed concentration bolus drug exposure. This fixed administration profile can be readily achieved in all research labs at present, but what has till now remained absent is the ability to replicate PK like drug concentrations profiles for humans (and animals) in microtiter plates, which are important factors in the successful development of a drug. One approach to testing compounds in vitro with varying concentrations has been the development of organ-on-chip multi-organ systems, which utilise the metabolic capability of cells often hepatocytes, to clear the compound from a system, creating concentration changes with time [29,30]. This approach, while closely mimicking physiology, is relatively complex requiring the simultaneous culture of multiple tissue types and is typically low throughput. We have designed an array of microfluidic pumps and valves that offer a drug exposure solution, which allows (1) individually addressable wells within a standard microtiter plate; (2) time division multiplex dosing of wells to generate PK profiles; (3) the ability to simultaneously deliver 2 drugs, each with their own PK profiles, to a single well (for combination studies), all using (4) a platform that fits within a standard incubator and is capable of running multiweek experiments. With cancer drug attrition rates being higher than other therapeutic areas, we chose to replicate the PK profiles of oncology compounds [20]. Here, we set out to demonstrate that the integration of microfluidics with multiwell plates enables the generation of PK like profiles in vitro for one or more drugs and that this facilitates prediction of mouse xenograft tumour responses as well as exploration of how alterations in PK parameters may influence drug efficacy.

The first study explored the efficacy of the ERK1/2 inhibitor AZD0364, where a difference in response of *KRAS* mutant Calu-6 and A549 xenograft models has recently been reported [23]. In vitro, the growth of Calu-6 and A549 cells were both significantly inhibited when manually dosed with a fixed concentration of AZD0364. This response was different to the reported in vivo efficacy, where Calu-6 xenografts are more sensitive than A549 xenografts to the same dose of AZD0364 [23]. In an effort to observe this difference in vitro, the Microformulator was used to replicate the in vivo profile of the daily dose of AZD0364 reported in the xenograft studies (50 mg/kg QD), in an in vitro experiment over a treatment period of 11 days. When treated with the Microformulator replicated profile, the growth of Calu-6 cells is still significantly inhibited by AZD0364, whereas the growth of the A549 cells is less impacted over the duration of this experiment. Thus, treating Calu-6 and A549 cells in vitro with this varying PK profile more accurately reflects the difference in sensitivity of the xenograft models to ERK1/2 inhibition and better replicates the in vivo compared to the fixed bolus concentration. This study demonstrated the capability of the Microformulator to replicate the in vivo profile and was not designed to interrogate whether standard in vitro techniques could obtain this sensitivity difference and if so at which concentrations or time points.

Secondly, we aimed to replicate the efficacy of a PARP inhibitor (olaparib) combined with a DNA-PK inhibitor. Chemotherapeutic drugs often lead to the formation of DNA double-strand breaks, which lead to genomic instability and cell death. Sensitivity to chemotherapeutic drugs can be increased through the inhibition of DNA-PK, a key enzyme in the nonhomologous end-joining DNA repair process [31,32]. AZD7648, a DNA-PK inhibitor, is an efficient sensitizer of radiation and chemotherapy-induced DNA damage and in combination with the PARP inhibitor olaparib increases genomic instability, ultimately resulting in cell growth inhibition/apoptosis [26]. Using the Microformulator to create olaparib and AZD7648 PK profiles with varied durations above IC_90_ in vitro resulted in tumour cell growth inhibition with similar rank order as observed with tumour xenografts. Specifically, the cell growth inhibition with Microformulator-replicated AZD7648 monotherapy compared well with tumour xenograft responses and in combination with olaparib the AZD7648 effects were enhanced. The Microformulator-replicated olaparib PK monotherapy condition gave greater efficacy in vitro than was observed in vivo; this may relate to greater tumour cell exposure in a 2D monolayer than in a 3D tumour xenograft or greater sensitivity to PARP inhibition. These results provide proof-of-concept that the Microformulator can replicate in vivo efficacy of oncology compounds in a cell-based assay by incorporating their in vivo exposure profiles.

The Microformulator is not limited to re-creating in vivo PK profiles but also could be used to investigate how theoretical changes in a drug exposure profile would influence a biological response. Insight gleaned from such studies could be used to optimise the dose and dosing schedule of a compound to enhance efficacy or alternatively, to minimise an undesired toxicological effect. Acquiring this type of information early in a drug discovery project would help define the desired profile of a candidate drug for clinical development. Importantly, this type of information can be generated in vitro with a compound that has sufficient cellular potency and does not require a more in vivo–optimised profile (oral bioavailability, metabolic stability, etc.) that typically occurs during the lead optimization phase. In the example with olaparib plus AZD7648, varying the length of DNA-PK target inhibition (5-, 14-, or 24-hour exposure) at a modest (IC_50_) or high level (IC_90_) of DNA-PK inhibition demonstrated that high target coverage for an extended period of time is required for maximal efficacy. This highlights the capability of the Microformulator to address specific questions with different combinations of concentrations and time coverage. Illustrating the potential to replicate known clinical or in vivo profiles, to mimic in silico profiles, or to execute completely new dosing strategies. Performing similar types of studies in mice may be technically feasible, but would be challenging, requiring for example surgically implanting minipumps for drug dosing, synthesis of significantly more compound and the generation of a drug formulation compatible with minipump use. Thus, Microformulator in vitro applications have additional advantages for data delivery and ultimately have the potential to reduce reliance on in vivo studies and thereby align with “3R” principles to reduce, replace, and refine the use of animal research studies in drug discovery [33,34].

In addition to the studies described above, the Microformulator has broader utility including the ability to perform additional experiments, for example, focused on efficacy/toxicity studies, enabling the understanding of acute dynamic toxicity responses. Observing the resultant effects as concentrations are modulated with time. Scheduled dosing and combination dosing studies are also possible, and a future direction of the ERK 1/2 inhibitor study above could explore AZD0364 in combination with other therapeutics such as MEK inhibitors, with the Microformulator enabling the scheduling of the different PK profiles in vitro. The device could extend applications further by incorporating PK exposures into more complex models utilising human primary cells or multi-cell type microtissues compatible with microtiter plates designed for spheroids/organoids or directly coupled to non-microtiter plate tissue holders including tissue chips and multiorgan models. Modifications to the device have been envisaged to simplify the design with a microfluidic manifold replacing the tubing, replacing the PDMS in pumps/valves for a material more resilient to small molecule absorption and encasing the device to protect it during use [35]. Minor modifications could also enable the use of non- or semi-adherent cells and Transwell plate formats that aspirate/deliver test compounds to either the basolateral or luminal compartments. Iterations such as these are likely to enable a more seamless integration into pharmaceutical workflows. Additionally, the Microformulator has the capability to replicate endocrine regulation and hormone circadian rhythms on in vitro cultures, an alternative to conventional practice of replacing media at defined intervals [36]. Furthermore, the continued long-term control over media within individual wells is likely to prove advantageous during parallel experiments to optimise stem cell differentiation protocols.

In conclusion, microtiter plates, with adherent cells and bolus addition of compounds, are integral to in vitro drug development, with large infrastructure and techniques developed around their use. Despite this, in vitro efficacy experiments using microtiter plates exhibit the key limitation of not replicating the drug concentration changes that happen inside of the body. Therefore, animal models are the gold standard when modelling compound efficacy for diseases such as cancer. There is a requirement to improve the translational results from in vitro models, and the Microformulator technology demonstrated here has the potential to be an important tool to improve the prediction of clinical efficacy, enabling more assured preclinical development decisions.

The Microformulator offers the advantages of rapid in vitro testing, compatibility with microtiter plates, achieving moderate throughput by creating distinct PK profiles within each well of a microtiter plate. Additionally, the device offers the opportunity to gain experimental evidence of the efficacy or otherwise of human PK predicted from animal or in silico studies. This is a significant gap in the current preclinical development paradigm. The device’s capabilities have been demonstrated by replicating drug PK profiles in vitro, resulting in outcomes not seen in conventional fixed bolus dosing. We also demonstrate the utility of the device to replicate single and drug combination PK profiles and reproduce the rank order of in vivo TGI in a microtiter plate.

The Microformulator offers a new approach to preclinical testing that targets a more predictive and efficient in vitro preclinical testing process for oncology compounds and beyond. This technology could improve standard in vitro techniques by adding another tool to the pharmacological toolbox.

## Materials and methods

### Microformulator—Components and system

The microfluidic device, referred to here as the Microformulator (Vanderbilt Institute for Integrative Biosystems Research and Education (VIIBRE), Vanderbilt University) is a system comprised of media bottles, computer-controlled microfluidic rotary planar peristaltic micropumps and rotary planar valves, tubing, and needles that enable real-time control of the formulation of media in individual wells within a microtiter plate (Fig 2). The device is split into 2 independent sides for simultaneous fluid addition and removal. On the dosing side, Tygon tubing (Cole-Parmer, USA) connects the input bottles to a 5-port valve, pump, a 25-port valve, and then to the individual blunt dosing needles situated above the liquid level within a well. The 5-port valve enables the selection of input media bottles, and the 25-port valve enables the selection of individual wells. The pump draws medium from the selected input media bottle (containing either media or drug stock solutions prepared in media), through the 5-port valve and to the selected port of the 25-port valve, allowing for the addition of media to an individual well. Similarly, on the aspiration side of the device, the 25-port valve selects the blunt needle, situated near the bottom of a well, and the pump draws medium from the well and into a waste bottle. All blunt needles are fixed in position 0.5 mm above the bottom of a well using a needle plate assembly lid, which ensures that wells are never left empty when fully aspirated.

The design, fabrication, and performance validation of the rotary planar valves and rotary planar peristaltic micropumps that comprise the Microformulator have been previously described [19] and were built using technologies developed at the VIIBRE. Other embodiments of the Microformulator and its pumps and valves have been described elsewhere [37,38]. Briefly, the valves and pumps were operated through the combination of stepper motor driven actuation and a polydimethylsiloxane (PDMS) microfluidic chip. A 25-port value was created to enable selecting fluid paths to each of 24 wells of a microtiter plate and thereby substantially increasing the throughput of the system (with the 25th port used to flush the common channel of the valve). Thus, the sequential combination of a 5-port valve, microfluidic pump, and a 25-port valve represents 1 microfluidic unit for dispensing media and drugs while a duplicate unit is used for aspiration. The system was applied to both 24-well microtiter plates (S3 Fig) as well as 1 quadrant of a 96-well microtiter plate. It has been demonstrated that the Microformulator can be expanded to simultaneously cover all 4 quadrants of an entire 96-well plate for higher throughput studies but was not required for the experiments performed in Figs 3–5. A valve placed after the aspiration pump would have the ability to direct media from the well into separate reservoirs; this sampling, however, was not required for experiments in Figs 3–5.

Stepper motors were controlled by a 4-channel USB microcontroller (MCU) (Arduino Uno R3, Arduino, USA), which was connected to a laptop and operated using Automated Multipump Experiment Running Environment software (AMPERE, VIIBRE) (S2 Fig). Controlling the speed and duration of operation of the pumps, the angular positioning of valves and the oscillations between sample inputs (media and drug stock solutions), AMPERE enabled controlled removal of media from wells and delivery of the desired drug concentrations into individual wells (flow rate = 2.4 +/− 1.7 to 890 +/− 77 μL/min) [19]. Flow rates of 400 to 750 μL/min were used in the experiments in Figs 3–5.

### Microformulator—Experimental PK preparation

Mouse PK data were modelled by fitting a one-compartment, first-order absorption model to available data across several studies covering a range of doses. The resulting PK data curves were split into predefined intervals; the median exposure value in each interval was used to define the target drug concentration for that step and was uploaded into the AMPERE control software. This created an AMPERE experimental file containing data necessary for the operation of the Microformulator, to perform concentration changes (S2 Fig). Concentration change frequency within a well was every 1 or 2 hours depending on the study and replicating specific single or multiple dose PK profiles over 24 hours, repeated daily for the length of the study.

### Microformulator—Assembly and operation

PDMS chips, tubing, blunt needles, and lids were steam sterilised at 121°C or above. Assembly was performed under aseptic conditions within a microbiological safety cabinet (Class II). Prior to experimentation microfluidic lines were flushed with ethanol and PBS. Media bottle inputs were made up with either vehicle media (diluent) or drug media (containing known concentrations of compounds). Media bottle lids were then connected to the input 5-port valve using embedded tubes in the sterile vented caps of the media bottles. An empty waste bottle was used for the aspiration/waste. The well plate containing the cell culture was inserted into the device. The needle plate housing, the blunt needles, and well plate were brought together. The device was then placed inside of an incubator (S3 Fig) and connected to a nearby laptop containing the AMPERE control software.

The experimental file was loaded into the AMPERE software and the operation of the device was started. The device dosed individual wells with varying compound concentrations at each step. For the completion of a single step in a well: Media was aspirated from the well through the aspiration side of the device; this could be performed in excess to empty up to the specified height of the blunt needle. On the dosing side of the device, a specified volume of blank media was then dosed into the well, followed by a specified volume of a compound/combination of compounds. This dilution of compound allows for control over the compound/s concentration at each step (Fig 2).

### Cell culture

FaDu ATM KO cells were generated at AstraZeneca [26]. Calu-6 and A549 cells were obtained from ATCC. All cell lines were routinely maintained in RPMI 1640 supplemented with 10% FCS, 1% L-glutamine, and 1% Penicillin–Streptomycin (Thermo Fisher, USA). Calu-6 and A549 cells were seeded at 10,000 cells per well of a 24-well plate (Costar, Corning, USA) and cultured within the Microformulator for up to 11 days. FaDu ATM KO cells were seeded at 1,500 cells per well in a 96-well plate for up to 21 days. Cells were allowed to equilibrate for 24 hours (FaDu) or 72 hours (Calu-6, A549) in a standard cell culture incubator (37°C and 5% CO_2_) before PK administrations commenced. Cell growth and confluency were monitored either using an IncuCyte ZOOM live cell analysis imaging system (Essen Bioscience, UK) or by analysis of brightfield microscopy images. Using the IncuCyte Zoom Cell confluency was determined from 4 fields of view per well every 24 hours over a period of 14 or 21 days. Brightfield images were captured throughout the experiment and confluency determined manually; results were verified separately with endpoint ATP quantification (Cell-TiterGlo, Promega, USA). DNA analysis was performed using Hoechst 33342 (1:5,000 dilution, 90-minute incubation at room temperature). Stained cell plates were read on a Cellomics ArrayScanTM VTI imaging platform (Thermo Scientific, USA), using an XF53 filter, 10× objective, with an LED light source to analyse nuclear staining with Hoechst 33342 (405 nM).

### SDS-PAGE and western blotting of samples

FaDu ATM KO cells were analysed for pharmacodyanamic biomarker modulation by western blotting. Protein lysates were generated by adding radioimmunoprecipitation assay (RIPA) lysis buffer supplemented with 1% sodium dodecyl sulfate (SDS) and protease and phosphatase inhibitor tablets (Roche, Switzerland) to FaDu ATM KO cells 2 hours following the start of compound treatment in the second 24-hour treatment cycle. Protein concentration was determined using the BCA protein assay kit (Thermo Fisher, USA). Proteins were separated by SDS-PAGE on NuPAGE 4% to 12% Bis-Tris (Thermo Fisher, USA) and transferred onto nitrocellulose membranes using the iBlot dry blotting system (Thermo Fisher, USA). Membranes were blocked with 5% milk in Tris-buffered saline (TBS) with 0.05% Tween-20 (TBS-T) and incubated with primary antibody overnight at 4°C. Primary antibodies used were anti-DNA-PKcs pS2056 (in-house generated), anti-DNA-PKcs (Cell Signalling, #12311, USA), and anti-vinculin (Sigma, 19191, USA). Membranes were then washed with TBS-T and incubated with HRP-conjugated anti-rabbit or anti-mouse antibodies for 1 hour at room temperature. Proteins were detected by incubating membranes with SuperSignal Dura extended-duration substrate (Thermo Fisher, USA) and visualised using the G:BOX ChemiGenius imaging system (Syngene, UK). Band intensities were quantified using Image J software.

### In vivo studies—Xenograft

FaDu ATM KO cells were cultured in MEM + 10% FBS, 1% L-Glutamine, and 1% NEAA at 37°C, 7.5% CO_2_. Female immunocompromised SCID (C.B-17/IcrHanHsd-Prkdcscid) mice (Envigo) were used for subcutaneous tumour implantation of these cells in serum free media at 5 × 10^6^ per mouse. AZD7648 was formulated in 0.5% hydroxypropyl methylcellulose/0.1% Tween80 (HPMC/T) and orally dosed (75 to 100 mg/kg). When dosed twice daily, the time between the morning and evening doses was 8 hours.

Olaparib was formulated in 10% DMSO/30% Kleptose and orally dosed at 50 mg/kg once daily. The combination was dosed 1 hour after the morning dose of AZD7648 or its vehicle HPMC/T. TGI from start of treatment was assessed by comparison of the mean change in tumour volume for the control and treated groups and represented as TGI.

In vivo studies were conducted in the UK in accordance with UK Home Office legislation, the Animal Scientific Procedures Act 1986, and AstraZeneca’s global bioethics policy. Experimental details are outlined in Home Office project licences 70/8839, 70/8894, and P0EC1FFDF. The AstraZeneca Animal Welfare Ethical Review Body “AWERB” reviewed and approved the study (Study Approval Number DNAPK1704). Euthanasia was performed using cervical dislocation followed by severing of the femoral vein for confirmation of death.

## Supporting information

S1 Fig Analysis of drug delivery using the Microformulator. (A) A drug-dispensing program was created in AMPERE to dispense 3-fold increases and then decreases in fluorescein concentration to cover a 3,000-fold concentration range. Actual fluorescein concentrations were quantified in each well using fluorescence intensity and compared to a fluorescein standard curve. (B) PK curves were created in AMPERE to deliver either AZD7648 or olaparib at 4-fold dilutions into separate microtiter wells. The actual concentration of compound in each well was quantified using mass spectrometry and compared to standard curves. Underlying data can be found in S4 Data. PK, pharmacokinetic.
(TIF)

S2 Fig Screen capture of the AMPERE software used to control the Microformulator throughout the experiment. On the left are cells, each row indicating 1 second of time within a specific device (column). These cells are used for scheduling experiments. On the right, the individual devices can be manually adjusted within the software for real-time use.
(TIF)

S3 Fig Photograph of the Microformulator. Addressing a 24-well microtiter plate inside the incubator.
(TIF)

S1 Data Data corresponding to Fig 3. (XLSX)

S2 Data Data corresponding to Fig 4. (XLSX)

S3 Data Data corresponding to Fig 5. (XLSX)

S4 Data Data corresponding to S1 Fig. (XLSX)

## Abbreviations:

DNA-PK: DNA-dependent protein kinase
KO: knockout
NSCLC: non-small cell lung cancer
PARP: Poly(ADP-Ribose) Polymerase
PD: pharmacodynamics
PK: pharmacokinetic
TGI: tumour growth inhibition
VIIBRE: Vanderbilt Institute for Integrative Biosystems Research and Education
