# Supplementary figures and images for: A microfluidic system that replicates pharmacokinetic (PK) profiles in vitro improves prediction of in vivo efficacy in preclinical models
Source: PLoS Biol. 2022 May 26;20(5):e3001624. doi: 10.1371/journal.pbio.3001624 (PMC9135222; doi:10.1371/journal.pbio.3001624)

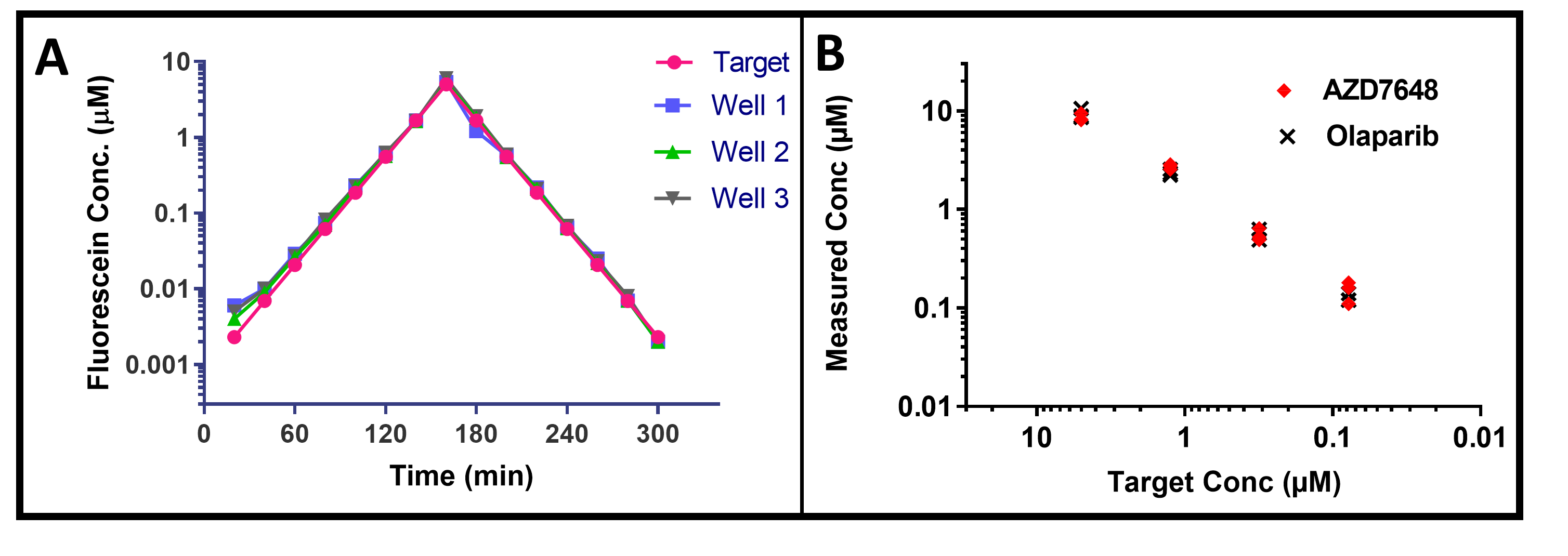

Supplement: S1 Fig — (A) A drug-dispensing program was created in AMPERE to dispense 3-fold increases and then decreases in fluorescein concentration to cover a 3,000-fold concentration range. Actual fluorescein concentrations were quantified in each well using fluorescence intensity and compared to a fluorescein standard curve. (B) PK curves were created in AMPERE to deliver either AZD7648 or olaparib at 4-fold dilutions into separate microtiter wells. The actual concentration of compound in each well was quantified using mass spectrometry and compared to standard curves. Underlying data can be found in S4 Data. PK, pharmacokinetic. (TIF) [file pbio.3001624.s001.tif]

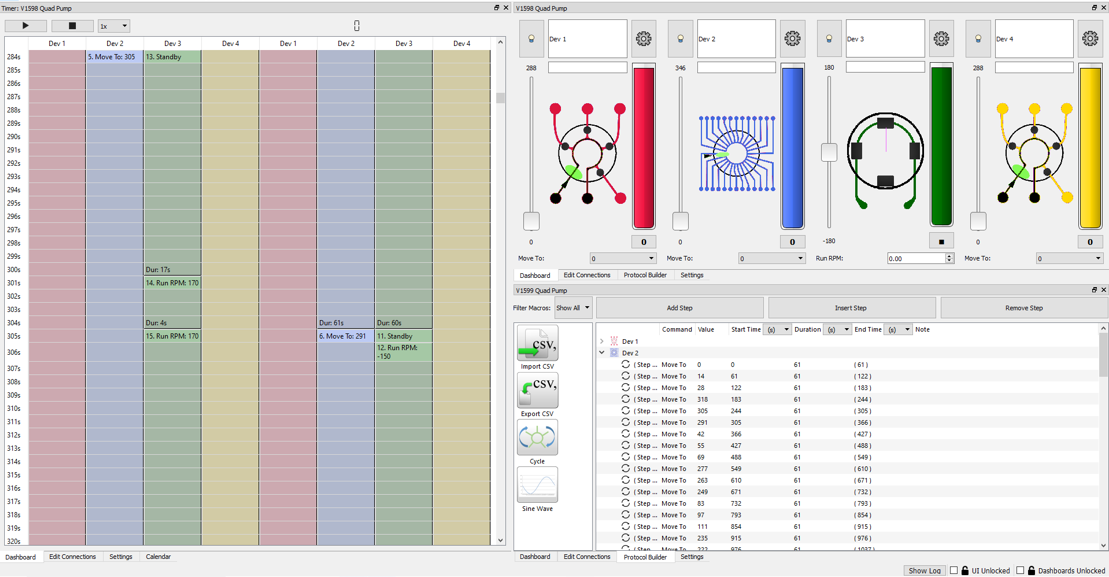

Supplement: S2 Fig — On the left are cells, each row indicating 1 second of time within a specific device (column). These cells are used for scheduling experiments. On the right, the individual devices can be manually adjusted within the software for real-time use. (TIF) [file pbio.3001624.s002.tif]

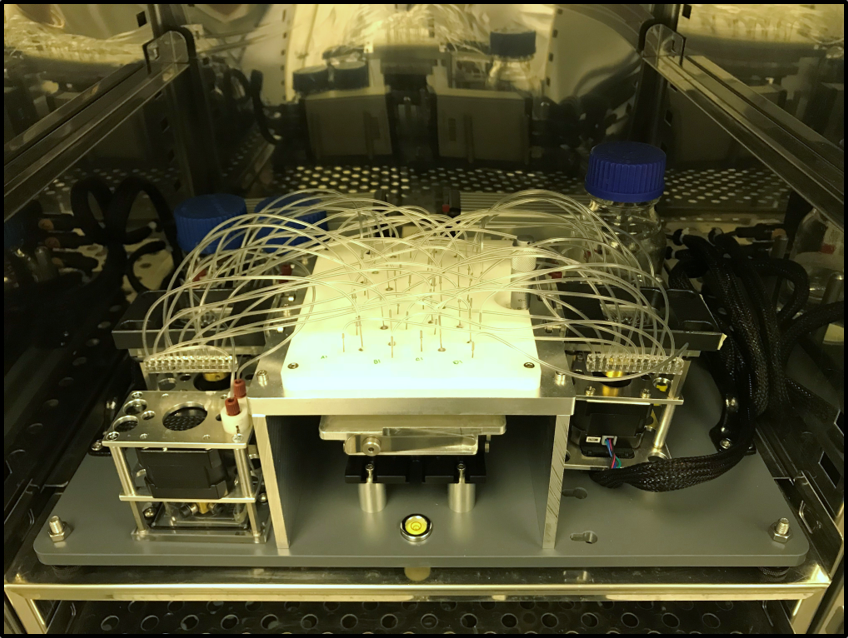

Supplement: S3 Fig — Addressing a 24-well microtiter plate inside the incubator. (TIF) [file pbio.3001624.s003.tif]
